# Immunopathogenesis of HIV Infection in Cocaine Users: Role of Arachidonic Acid

**DOI:** 10.1371/journal.pone.0106348

**Published:** 2014-08-29

**Authors:** Thangavel Samikkannu, Kurapati V. K. Rao, Hong Ding, Marisela Agudelo, Andrea D. Raymond, Changwon Yoo, Madhavan P. N. Nair

**Affiliations:** 1 Department of Immunology, Institute of NeuroImmune Pharmacology, Herbert Wertheim College of Medicine, Florida International University, Modesto A. Maidique Campus, Miami, Florida, United States of America; 2 Department of Biostatistics, Robert Stempel College of Public Health and Social Work, Florida International University, Modesto A. Maidique Campus, Miami, Florida, United States of America; University of Nebraska Medical Center, United States of America

## Abstract

Arachidonic acid (AA) is known to be increased in HIV infected patients and illicit drug users are linked with severity of viral replication, disease progression, and impaired immune functions. Studies have shown that cocaine accelerates HIV infection and disease progression mediated by immune cells. Dendritic cells (DC) are the first line of antigen presentation and defense against immune dysfunction. However, the role of cocaine use in HIV associated acceleration of AA secretion and its metabolites on immature dendritic cells (IDC) has not been elucidated yet. The aim of this study is to elucidate the mechanism of AA metabolites cyclooxygenase-2 (COX-2), prostaglandin E2 synthetase (PGE_2_), thromboxane A2 receptor (TBXA_2_R), cyclopentenone prostaglandins (CyPG), such as 15-deoxy-Δ12,14-PGJ2 (15d-PGJ2), 14-3-3 ζ/δ and 5-lipoxygenase (5-LOX) mediated induction of IDC immune dysfunctions in cocaine using HIV positive patients. The plasma levels of AA, PGE_2,_ 15d-PGJ2, 14-3-3 ζ/δ and IDC intracellular COX-2 and 5-LOX expression were assessed in cocaine users, HIV positive patients, HIV positive cocaine users and normal subjects. Results showed that plasma concentration levels of AA, PGE_2_ and COX-2, TBXA_2_R and 5-LOX in IDCs of HIV positive cocaine users were significantly higher whereas 15d-PGJ2 and 14-3-3 ζ/δ were significantly reduced compared to either HIV positive subjects or cocaine users alone. This report demonstrates that AA metabolites are capable of mediating the accelerative effects of cocaine on HIV infection and disease progression.

## Introduction

During the last decade, an intertwined epidemic of drug abuse and HIV-1 infections has emerged. Globally there were an estimated 34.2 million people living with HIV [1]. Illicit drug abuse including cocaine is a significant risk factor for HIV infection and AIDS disease progression [2], [3]. Cocaine is currently being used worldwide in epidemic proportions, particularly in the U.S. The 2010 report shows that 1.5 million Americans (aged 12 or older) are cocaine users [4]. Overall, about 16 million injecting drug users are present worldwide and 3 million (18.9 per cent) of them are living with HIV [1]. Previous studies suggest that cocaine use and HIV-1 infection are independently associated with immune dysfunction which leads to neuronal impairments [5], [6].

Dendritic cells (DC) play a significant role as the first line of defense against viral pathogens and illicit drug effects [7], [8]. HIV-1 directly affects dendritic cells (DC) and leads to dysfunction of immune system manifested by increased levels of inflammatory cytokines, chemokines and neurotoxin such as quinolinic acid and arachidonic acid (AA) [9], [10]. Increasing evidence suggests that DCs play a major role in the defense against HIV infection and illicit drug such as cocaine [11]–[13]. Immature dendritic cells (IDC) specialize in capturing and processing antigens and plays wide role in cell maturation, migration to CD4+ T cells, and T cell activation [14]. Previous studies indicate that AA metabolites such as COX-2, TBXA2, 5-LOX and 15d-PGJ2 found in specific DC subsets interplay with immune regulation [15], [16]. Also, AA metabolites COX-2 induce T-cell tolerance to antigenic stimuli which could affect immune functions [17]. Indeed, expression of COX-2 activation subsequently affect via TBXA2, 15d-PGJ2 and 5-LOX which are the potential markers of viral replication as well as immune and neuronal impairments [18], [19]. However, the COX-2 and 5-LOX can be regulated via monocytes and dendritic cells through activation of T cells signaling during inflammatory processes [20]. Furthermore, the 5-LOX enzyme plays an important role in leukotriene B_4_, a potent inflammatory mediator in peripheral disorders [21], and neurotoxicity [22]. The members of the PGJ2 class, 15d-PGJ2 (also called cyclopentenone PGs, CyPG), play a role in checkpoint of cytokine/chemokine synthesis and intracellular translocation of HIV viral protein and viral replication [23]. 15d-PGJ2 has anti-inflammatory properties [24], and it negatively regulates PGE_2_ synthetase. However, increased levels of AA directly bind with 14-3-3 ζ/δ protein polymerization and affect their cellular function [25]. Furthermore, decreased 14-3-3 ζ/δ proteins subsequently affect platelet aggregation mediated by platelet activating factor (PAF), which may induce apoptosis.

Studies have consistently demonstrated that cocaine use and HIV infection accelerates viral replication, disease progression which leads susceptibility and severity of immune dysfunction [3], [25] which leads to HIV-associated neurocognitive disorder (HAND) [26]. HIV positive cocaine user’s exhibit accelerated disease progression compared to non- cocaine using HIV positive individuals [2, 3. 26, 27]. Our recent report demonstrated that HIV derived gene product gp120 with cocaine interaction potentiated the additive effect of AA metabolite COX-2 induction in primary astrocytes [28]. Despite mounting evidence which suggests that cocaine use may exacerbate HIV disease, mechanistic studies assessing the interactive role of cocaine and HIV infection on DC and their role remains to be determined.

In this study, we investigated the role of AA metabolites COX-2, PGE_2_, 15d-PGJ2, 14-3-3 ζ/δ, TBXA_2_R, and 5-LOX associated immunopathogenesis in HIV positive cocaine users. We showed that cocaine use in HIV positive subjects enhances immune dysfunction and exacerbates the neurotoxin AA metabolites gene, protein and intracellular expression in IDC.

## Methods and Materials

### Human subjects

Blood donors were recruited from the Borinquen Health Care Center, Inc., Miami. Written and signed consent forms were obtained from all participants in compliance with Florida International University (FIU) and the National Institutes of Health (NIH) policies. All participants consent forms are kept in a lock cabinet to maintain confidentiality. The protocol was approved by the IRB of FIU. Peripheral blood from normal, HIV positives, cocaine users and HIV-1 positive cocaine users was drawn into heparin lined tubes (20cc). Human subjects were recruited through collaborating physicians, community agencies, as well as by participant referral. After eligibility was confirmed, informed consents were obtained. Participants provided medical documentation to confirm HIV and Hepatitis B and C status. For HIV positive individuals, medical documentation confirmed CD4 and Viral loads. Individuals younger than 18 years old and with Hepatitis B and/or Hepatitis C infection were deemed ineligible. Self-report data was collected concerning drug use history, and participant’s blood specimens were collected by a Registered Nurse.

### HIV-infected and control populations

HIV-1 infection are defined as individuals who exhibit a faster decline in CD4 lymphocytes, displaying a declining slope of >50 CD4 T cells per cubic millimeter per semester and an overall CD4 loss >30% with a current CD4 count <500 and plasma viral load >50,000 copies/ml. However, in the present study, overall patients were taking more than two antiretroviral drugs and the patients taking ART showed increase in CD4 significantly. The number of normal subjects, cocaine user, HIV positive and HIV positive cocaine user average age and CD4 counts are given in Table 1. Cocaine use with/without HIV were confirmed by urine toxicology testing on the day of blood collection. Control or normal groups (drug free and HIV negative) were healthy volunteers who were age-, sex-, and ethnically matched with HIV-1-infected subjects and cocaine users. A clinical history was obtained from all normal donors. However, in this study only cocaine user and HIV positive cocaine users were recruited. Exclusion criteria for all groups were age <18 and >50 years, pregnancy, Hepatitis B and C (Table 1).

**Table 1:** Baseline characteristics of HIV positive subjects with cocaine user.

	Normal Subjects	Cocaine User	HIV Positive	HIV Positive Cocaine user
Age	32.46±6.22	41.52±3.81	36.14±3.75	44.57±4.63
Sex	Male –9 Female – 3	Male –6 Female – 6	Male –11 Female - 8	Male –9 Female – 9
CD4	–	–	≥466/µl	≥308/µl

### Isolation and generation of immature DC (IDC)

DCs were prepared from PBMC as described [2]. Blood samples from normal, cocaine users, HIV positive and HIV positive cocaine users were collected and Peripheral Blood Mononuclear Cells (PBMC) were separated on a density gradient and adhered to plastic culture plates in serum containing medium. Non adherent cells were removed after 1 h at 37°C, and adherent cells were cultured for 6 days in medium containing 100 U/ml recombinant human GM-CSF and 100 U/ml IL-4 (R&D Systems). After 6 days of culture, iDC were removed by gently swirling the plate to resuspend them for use in the experiments. IDC were washed in FACS buffer (eBioscience), incubated with nonspecific IgG (20 µg/ml) for 10 min at 4°C to block FcR, stained with specific Abs for DC surface markers, and analyzed by flow cytometry. The IDC express CD80, CD86, CD40, HLA-DR, DQ, and CD11c at different levels.

### RNA Extraction and Real time quantitative PCR (qRT-PCR)

Total RNA from IDC was extracted using the Qiagen kit (Invitrogen Life Technologies, Carlsbad, CA, USA) following the manufacturer’s instructions. The total RNA (3 µg) was used for the synthesis of the first strand of cDNA. The amplification of cDNA was performed and using specific primers for COX-2 (Assay ID, Hs00153133), TBXA2 R (Assay ID, Hs00169054), 5-LOX (Assay ID Hs00386528), and β-actin (Assay ID, Hs99999903) (Applied Biosystems, Foster City, CA) was used as housekeeping gene for quantifying real-time PCR. Relative abundance of each mRNA species was assessed using brilliant Q-PCR master mix from Stratagene using Mx3000P instrument which detects and plots the increase in fluorescence versus PCR cycle number to produce a continuous measure of PCR amplification. Relative mRNA species expression was quantitated and the mean fold change in expression of the target gene was calculated using the comparative CT method (Transcript Accumulation Index, TAI  = 2^−ΔΔCT^). All data were controlled for quantity of RNA input by performing measurements on an endogenous reference gene, β-actin. In addition, results on RNA from treated samples were normalized to results obtained on RNA from the control, untreated sample.

### Quantification of PGE_2_ and 15d-PGJ2 by enzyme- linked immunosorbent assay (ELISA)

Plasma were separated from normal, cocaine users, HIV positive patients, and cocaine using HIV positive subjects as previously mentioned. Plasma was analyzed for PGE_2_ (GenWay Biotech Inc. San Diego, CA) and 15d-PGJ2 (Enzo Life Sciences, Farmingdale, NY) using commercially available ELISA kits as per the manufacturer’s instructions.

### Level of neurotoxin AA in plasma by GC/MS

Blood samples were centrifuged at 2400 *g* for 10 min at 4°C. Aliquots (1 mL) of plasma were transferred to Eppendorf tubes and were stored at –70°C until analysis. Plasma 50 µl was hydrolyzed in the presence of 1 mol/L potassium hydroxide at 40°C for 30 min. 50 µL of distilled water, 3 µL of concentrated hydrochloric acid, 200 µL of ice-cold Folch solution (chloroform–methanol, 2∶1 by volume), and 50 ng of AA-d_8_ (internal standard) were added to 50 µL of hydrolyzed sample. After thorough vortex-mixing, the sample was centrifuged at 2400 *g* for 5 min. The lower organic layer was transferred to another Eppendorf tube and evaporated under nitrogen. The residue was then dissolved in 100 µL distilled water, and AA was extracted by the addition of 100 µL of hexane. The hexane extracts were dried under nitrogen and AA was analyzed by GC/MS, according to the modified method of Hadley et al [29].

### Analysis of intracellular expression of COX-2 and 5-LOX in IDC

Cocaine and HIV induced intracellular COX-2 and 5-LOX expressions were analyzed by flow cytometry in FACS caliber (BD Bioscience, San Jose, CA). Briefly, IDC (5×10^5^) were separated from normal, cocaine users, HIV positive patients, and HIV positive with cocaine user’s subjects. Cells were harvested and washed twice with PBS. The cells were fixed and permeabilized by Cytofix/Cytoperm Kit (BD Pharmingen, San Diego, CA, USA) following the manufacturer’s instructions. Cells were incubated with anti-COX2 (FITC) and anti-5 LOX (FITC) (antibodies-online, Atlanta, USA) for 20 min at 4° than washing with PBS and analyzed intracellular COX-2 and 5-LOX expression.

### Western blot analysis

To determine the COX-2, TBXA2 R and 5-LOX protein modification in IDC and serum 14-3-3 ζ/δ protein levels in normal, cocaine users, HIV positive and cocaine using HIV positive subjects. Equal amount of total cellular protein were resolved on a 4–15% gradient polyacrylamide gel electrophoresis, transferred to a nitrocellulose membrane and incubated with their respective primary antibodies. Immunoreactive bands were visualized using a chemiluminescence western blotting system according to the manufacturers’ instructions (Amersham Piscataway, NJ, USA).

### Data analysis

In general total of 60 samples were utilized for the experiments. However, some of the experiments as indicated in the figures at least six samples were utilized in each group. The values obtained were averaged and data are represented as the mean ± standard error. All the data were analyzed using GraphPad Prism- software version 5. Comparisons between groups were performed using one-way ANOVA and differences were considered significant at p≤0.05 and we performed post- test using “t” test.

## Results

### Demographics of Cohort

A total of 60 subjects were utilized for the study. The groups were as follows: 12-control (normal) subjects, 12- cocaine users, 19- HIV positive subjects and 18- HIV positive subjects with long-standing history of cocaine use. The groups of patients and control subjects were similar in age with male predominance.

### Cocaine increases HIV-1induced AA metabolites in IDC

**Figure 1:**
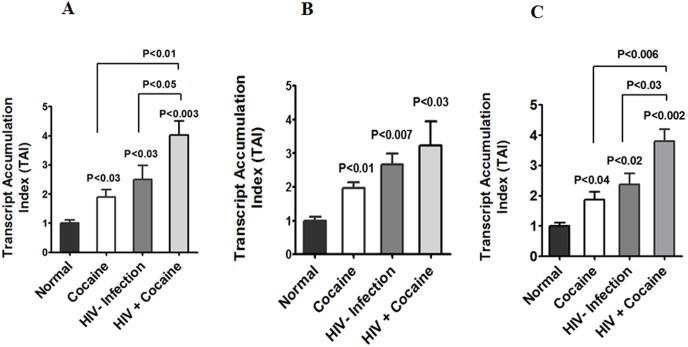
Effect of arachidonic acid metabolites COX-2, TBXA2 R and 5-LOX gene expression. IDC (3×10^6^ cells/ml) were isolated from normal, cocaine users, HIV positives and HIV positive cocaine users. RNA was extracted and reverse transcribed followed by quantitative real time PCR for COX-2 (A), TBXA2 R (B), 5-LOX (C), and housekeeping β-actin specific primers. Data are expressed as mean ± SD of TAI values of six independent human samples.

Studies have shown that HIV positive subjects who have used illicit drugs are more sensitive to immune-neuropathogenesis than either substance abusers or HIV positive subjects [26], [30]. In this study, we have investigated the possible role of cocaine increasing HIV induced AA and its metabolites COX-2, TBXA_2_ and 5-LOX in IDC. Data presented in Fig. 1A show IDC from cocaine users (p<0.03), HIV positive patients (p<0.03) and cocaine users with HIV positive subjects (p<0.003) significantly upregulated COX-2 gene expression compared to normal subjects. Fig. 1B shows a significant increase in TBXA_2_ gene expression cocaine users (p<0.01), HIV positive (p<0.007) and cocaine user with HIV positive subjects (p<0.03) compared with normal subjects. To examine whether AA metabolites of 5-LOX expression are similar or different from COX-2 expression, we performed expression profiles of 5-LOX. Fig. 1C results indicate that in cocaine users (p<0.04), HIV positives (p<0.02) and cocaine users with HIV positive subjects (p<0.002) have significantly higher levels of 5-LOX gene expression, compared to normal subjects.

### Cocaine impact of AA metabolites PGE_2_ and 15d-PGJ2 in HIV-infected Plasma

**Figure 2:**
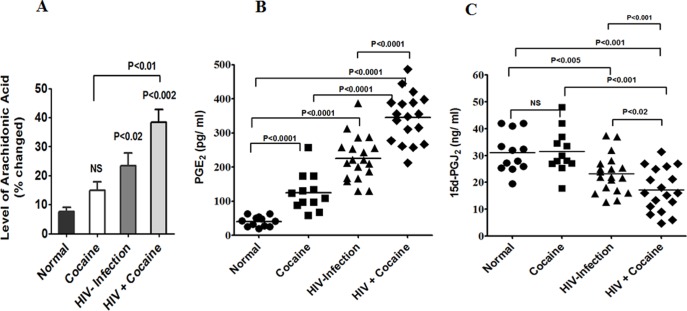
The level of AA, PGE_2_ and 15d-PGJ2 in cocaine users and HIV infected subjects. The plasma was separated and analyzed AA by GC/MS, PGE_2_ and 15d-PGJ2 level by ELISA in normal, cocaine users, HIV positives, and HIV positive cocaine users. Data are expressed as % fold change of AA (A), and ng/ml plasma (B and C).

Our previous report demonstrated that AA metabolites potentiated additive effect of gp120 with cocaine [28]. Therefore, in this study we also examined the level of AA, metabolites PGE_2_ and 15d-PGJ2 in cocaine users, HIV positives and cocaine users with HIV positives subjects. Data presented in Fig. 2A show that cocaine users have increased levels of AA but not significant; however, HIV positive (p<0.02) and cocaine users with HIV positives (p<0.002) have significantly increased higher levels of AA compared to normal subjects. The level of PGE_2_ in cocaine users and HIV positive cocaine users shows a significant increase when compared to controls. The data in Fig. 2B show the level of PGE_2_ respectively in HIV positive subjects (p<0.0001), cocaine user (p<0.0001) and cocaine users with HIV positive subjects (p<0.0001). Cocaine and HIV infection is synergistic as evidenced by significantly increased PGE_2_ (p<0.0001) when compared to either HIV positive (p<0.0001) or cocaine use alone (p<0.0001). In addition, the AA metabolite of anti-inflammatory 15d-PGJ2 is the major player in controlling the onset and resolution of acute inflammation. Since no studies have reported on the quantification of 15d-PGJ2 in HIV infected drug abusers. Fig. 2C indicated that the level of 15d-PGJ2 in HIV positives cocaine users shows a significant decrease (P<0.02) when compared to either cocaine or HIV positive (P<0.02) subjects. In the analysis of present data using in ANOVA the values were significantly less than p<0.001 and accordingly suggest the synergistic effects in the anti-inflammatory response which leads to increased AA metabolites in HIV positive cocaine users.

### Cocaine accelerates intracellular expression of COX-2 and 5-LOX

**Figure 3:**
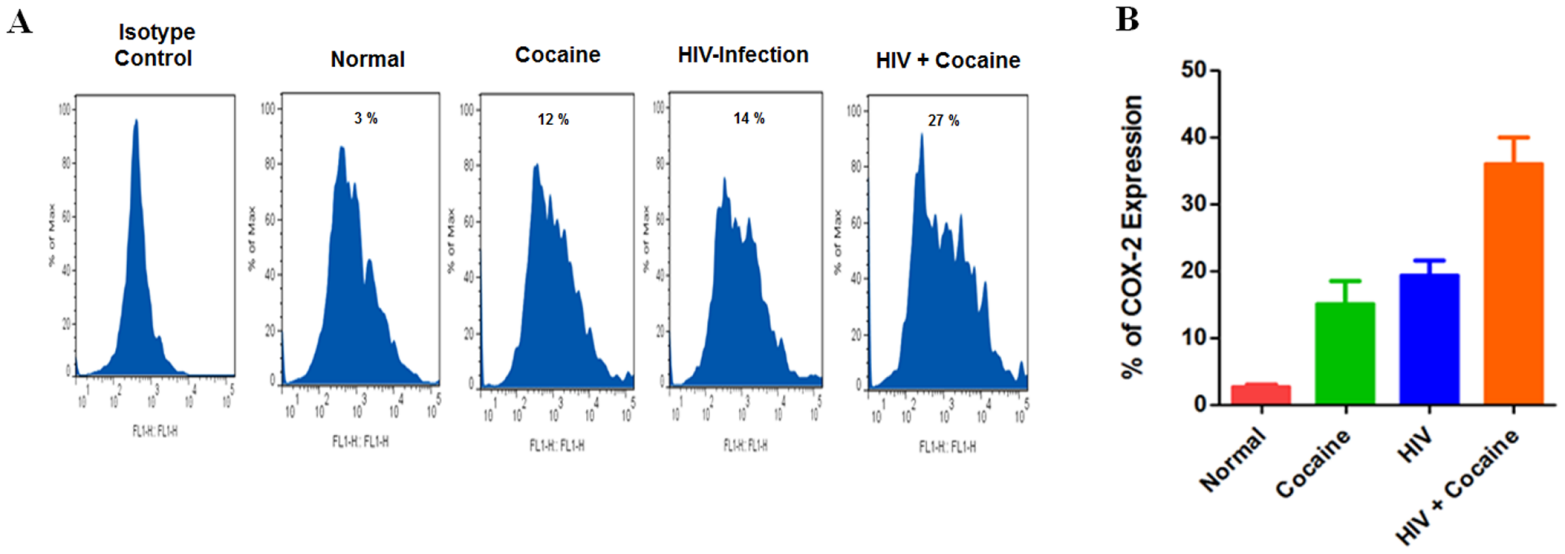
Effect of intracellular COX-2 expression in cocaine users and HIV infected patients. IDC (5×10^5^ cells/ml) were isolated from normal, cocaine users, HIV positive and HIV positive cocaine user intracellular expression of COX-2 (A) and % COX-2 positive cells (B) was analyzed by flow cytometry. Data are expressed and represented as mean ± SD of six independent experiments.

**Figure 4:**
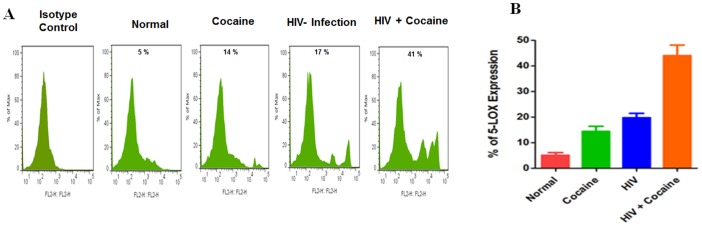
Effect of intracellular 5-LOX expression in cocaine users and HIV infected patients. IDC (5×10^5^ cells/ml) were isolated from normal, cocaine users, HIV positives and HIV positive cocaine users intracellular expression of 5-LOX (A) and % 5-LOX positive cells (B) was analyzed by flow cytometry. Data are expressed and represented as mean± SD of six independent experiments.

Since increased AA levels might results in modulation of intracellular levels of COX-2 and 5-LOX, and these two important enzymes are metabolized polyunsaturated fatty acids that function as parallel pathways on AA metabolism, we analyzed intra cellular expression levels of COX-2 and 5-LOX by flow cytometry. Data presented in Fig. 3 shows COX-2 expression in cocaine user (12%), HIV positive (14%) and HIV positive with cocaine users (27%) compared with control. Fig. 4 demonstrated that 5-LOX expression significantly increased in HIV positive cocaine users (41%) in comparison with either cocaine users (13%) or HIV positive subjects (17%) alone. These results suggest that HIV positive cocaine users have higher levels of AA and metabolites of COX-2 and 5-LOX expression.

### Cocaine exacerbates HIV-induced protein modification in IDC

**Figure 5:**
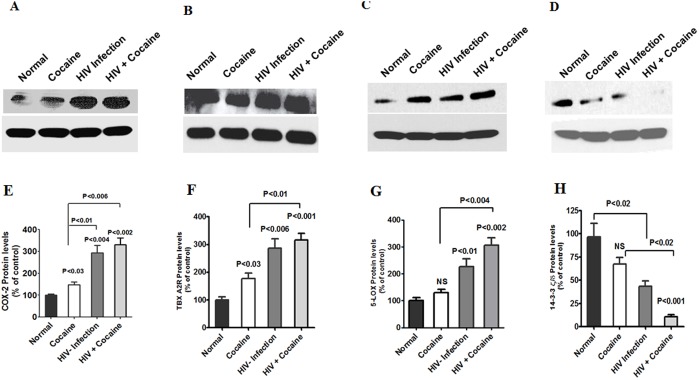
Effect of arachidonic acid metabolites COX-2, TBXA2 R and 5-LOX protein expression in IDC and 14-3-3 δ/ζ in plasma were isolated from normal, cocaine user, HIV positives and HIV positive cocaine users. Equal amount of IDC cell lysate and plasma protein were resolved by 4–15% SDS-PAGE and protein expression were analyzed by Western blot showing COX-2 (A), TBXA2 R (B), 5-LOX (C) and 14-3-3 δ/ζ (D). Figure E, F, G and H represented % densitometric values of COX-2, TBXA2 R, 5-LOX and 14-3-3 δ/ζ protein levels (% control). Data are expressed as mean ± SD of three independent experiments.

In order to understand protein expression mimics similar to gene expression in AA metabolites in HIV positive cocaine users. Therefore, we also analyzed COX-2, TBXA_2_ R, 5-LOX and 14-3-3 ζ/δ protein expression in terms of cocaine users, HIV infected subjects and cocaine using HIV infected patients in IDC. Fig. 5 demonstrates that COX-2 (A), TBXA_2_ R (B) and 5-LOX (C) protein is significantly up regulated whereas 14-3-3 ζ/δ (D) significantly reduced by cocaine users, HIV positive subjects and HIV positive with cocaine users. These observed results are consistent with cocaine using HIV infected patients levels of AA and metabolites PGE_2_ and 15d-PGJ2_._ Data presented in Fig. 5 E, F, G and H show the densitometry evaluation respectively COX-2 (p<0.009), TBXA_2_ R (p<0.009), 5-LOX (p<0.004) and 14-3-3 ζ/δ (p<0.007). These results confirm increased level of AA, PGE_2_ and COX-2 expression subsequently decreased anti-inflammatory level of 15d-PGJ2 and 14-3-3 ζ/δ protein inhibition in cocaine users, HIV positive subjects and HIV positive cocaine users when compared with normal subjects.

## Discussion

Previous studies have shown that AA and its metabolites play a wide role in immune dysfunction, behavioral impairments as well as viral replication and disease progression in HIV-infection and substance abuse [9], [18], [23]. The increasing AA metabolites COX-2 and 5-LOX are associated with HAD or HAND. COX-2 enzyme is an important player in the regulation of immune functions (e.g. immune tolerance) of antigen-presenting cells such as macrophages or DC [31], [32]. An increase in AA secretion by HIV infection and their metabolites COX-2, PGE_2_, TBXA_2_ and 5-LOX are found in cerebrospinal fluid (CSF) of HAD-patients [33]–[35]. However, overstimulation of AA leads to increase in its metabolites COX-2 and PGE_2_ [36], [37], and subsequently decreased the level of 15d-PGJ2 and 14-3-3 ζ/δ, which may play a vital role in immune dysfunction and disease progression [38], [39] in HAD patients [26]. The DC differentiation, maturation, migration, and antigen presentation function are modulated by COX-2 induced prostaglandins (PGs) [15], [40], [41]. However, there are no reports on the impact of cocaine on AA metabolites in HIV positive cocaine users. The present study provides new insights into the functional role of COX-2 in AA metabolites TBXA2 which subsequently affects 5-LOX in HIV infection and cocaine use. Our previous *in vitro* study has shown that the HIV-1 gp120 protein induces the COX-2 mRNA expression and protein modification implicated in neuro-AIDS [42].

In the present study, we have demonstrated for the first time that cocaine users, HIV positive and HIV positive cocaine users have increased levels of AA and mRNA expression of metabolites COX-2, TBXA2, 5-LOX (Fig. 1), and the levels of AA and PGE_2_ (Fig. 2) are associated with reduction in 15d-PGJ2 compared to normal subjects. It is known that AA metabolites PGE_2,_ COX-2, TBXA_2_ and 5-LOX are the major players in immune dysfunction [43]–[45], and reduced level of 15d-PGJ2 and 14-3-3 ζ/δ, may enhance viral replication and disease progression. These studies suggest that cocaine abusing HIV positive subjects may have an enhanced role of COX-2 and AA metabolites compared to normal subjects. This is consistent with earlier reports of gp120 induced neuroblastoma cells and HIV infected pulmonary hypertension, where activation of the COX-2 and 5-LOX pathways has been observed [36], [46]. Also, studies have shown that suicidal behavioral impairments are associated with 5-LOX increased in cerebral cortex brain regions [47]. These studies further confirm that the downstream effect of TBXA2 and 5-LOX are upregulated in cocaine using HIV positive subjects leading to depression and suicidal behavior. However, TBXA2 is an unstable product which initiates the PAF, blood aggregation induction and subsequently increases TBX B, which may be mediated by 14-3-3 ζ/δ leading to immune and neuronal impairments. Supporting our hypothesis, recent studies have demonstrated that 14-3-3 ζ/δ protein play a wide role in cytoskeletal translocation and platelet dysfunctions [48]. These results confirm that increased AA metabolites exacerbate the immune dysfunction in cocaine abusing HIV infected subjects.

Further, our results show that in cocaine users, HIV infected subjects, and cocaine using HIV infected subjects induction of intracellular COX-2 and 5-LOX expression (Fig. 3 and 4) is associated with a concomitant activation of COX-2 and 5-LOX protein (Fig. 5). The main observation in this report is that HIV positive and cocaine using HIV positive subjects have higher levels of PGE_2_ due to secretion of AA and COX-2 activation and subsequently reducing the level of 15d-PGJ2 and 14-3-3 ζ/δ. However, HIV positive cocaine users have higher levels of AA and metabolites. This suggests that HIV positive cocaine synergistically potentiate disease progressive effect when compared to either cocaine use or HIV positive alone.

Previous studies indicate that AA metabolites affect monocytes in HIV positive drug users [33] and HIV-1 envelop protein gp120 induced viral replication subsequently affect the immune function in human DCs [40]. In the present study, IDC in cocaine using HIV infected subjects showed an increased level of AA metabolites COX-2, TBXA2 R and 5-LOX gene expression and protein modification with significant increase of PGE_2_ levels compared to normal subjects. Also, increased functional COX-2 enzyme activity in DC could lead to an enhanced production of neurotoxin AA [49], which alters immune tolerance and causes imbalance resulting in immuno-neuropathogenesis [50]. These results suggest that IDC play a role in protective mechanisms of immune function, during HIV infection and cocaine abuse since both can alter AA levels and subsequently accelerate disease progression mediated by COX-2 and 5-LOX.

Overall, the data provide evidence of an interaction of cocaine use and HIV infection leading to an association between AA and its metabolites COX-2, TBXA2 R and 5-LOX by increasing the levels of PGE_2_, and formation of neurotoxin AA subsequently reducing the levels of 15d-PGJ2 and 14-3-3 ζ/δ in HIV positive cocaine users. Based on these results, AA and its metabolites potentiate the HIV disease progression by impairing the possible immune function [3], [26], [27], [51].
